# Disease Modelling of Cognitive Outcomes and Biomarkers in the European Prevention of Alzheimer’s Dementia Longitudinal Cohort

**DOI:** 10.3389/fdata.2021.676168

**Published:** 2021-08-20

**Authors:** James Howlett, Steven M. Hill, Craig W. Ritchie, Brian D. M. Tom

**Affiliations:** ^1^ MRC Biostatistics Unit, University of Cambridge, Cambridge, United Kingdom; ^2^ Centre for Clinical Brain Sciences, University of Edinburgh, Edinburgh, United Kingdom

**Keywords:** Alzheimer’s disease, biomarkers, cognitive functioning, disease modelling, European prevention of Alzheimer’s dementia, latent class mixed models, precision medicine, Bayesian profile regression

## Abstract

A key challenge for the secondary prevention of Alzheimer’s dementia is the need to identify individuals early on in the disease process through sensitive cognitive tests and biomarkers. The European Prevention of Alzheimer’s Dementia (EPAD) consortium recruited participants into a longitudinal cohort study with the aim of building a readiness cohort for a proof-of-concept clinical trial and also to generate a rich longitudinal data-set for disease modelling. Data have been collected on a wide range of measurements including cognitive outcomes, neuroimaging, cerebrospinal fluid biomarkers, genetics and other clinical and environmental risk factors, and are available for 1,828 eligible participants at baseline, 1,567 at 6 months, 1,188 at one-year follow-up, 383 at 2 years, and 89 participants at three-year follow-up visit. We novelly apply state-of-the-art longitudinal modelling and risk stratification approaches to these data in order to characterise disease progression and biological heterogeneity within the cohort. Specifically, we use longitudinal class-specific mixed effects models to characterise the different clinical disease trajectories and a semi-supervised Bayesian clustering approach to explore whether participants can be stratified into homogeneous subgroups that have different patterns of cognitive functioning evolution, while also having subgroup-specific profiles in terms of baseline biomarkers and longitudinal rate of change in biomarkers.

## 1 Introduction

Alzheimer’s disease (AD), the leading cause of dementia globally (Livingston et al., 2017), is characterised by synaptic dysfunction and neurodegeneration (e.g., neuronal loss), triggered by sequential accumulation of amyloid plaques and neurofibrillary tangles (aggregates of hyperphosphorylated tau proteins) (Braak and Braak, 1991). The exact ordering of the pathological cascade of events, leading to clinical symptoms of cognitive deterioration and dementia, has been actively researched over the last decade. Jack and colleagues (Jack et al., 2010; Jack et al., 2013) hypothesised that there is an underlying disease process and that the temporal ordering of changes in key biomarkers and their dynamics characterise the full spectrum of the disease throughout the different successive stages of pre-clinical, prodromal and dementia.

On the whole there have been few treatment successes (and none of these are disease-modifying) despite substantive investment in pharmacological compounds for Alzheimer’s disease in symptomatic populations and early promise shown in pre-clinical studies (Gauthier et al., 2016; Winblad et al., 2016; Anderson et al., 2017). There may be a number of possible explanations for the many failures including inadequate drug dosages, incorrect treatment targets and inappropriate trial populations where the disease process is too far along to be amenable to treatment (Raket, 2020; Shi et al., 2020; Yiannopoulou and Papageorgiou, 2020). There is a consensus that the genesis of AD pathology occurs decades before the onset of dementia symptoms (Braak and Braak, 1997; Hardy and Selkoe, 2002; Jack et al., 2010; Bateman et al., 2012; Braak and Del Tredici, 2012; Jack et al., 2013). This thus presents an opportunity for early disease course modification before dementia onset and even prior to clinical symptoms. As such there is great interest—from both academia and industry—in accurately identifying groups of individuals with higher likelihood of progressing to AD dementia for natural history studies, early phase treatment trials and for participation in secondary prevention trials where, for example, they may have evidence of AD pathology through relevant biomarker abnormalities but no clinical evidence of symptoms of dementia (Ritchie et al., 2016; Watts, 2018).

Current proposals for defining an individual’s probability for developing AD dementia or for modelling cognitive deterioration based on biomarkers and/or clinical symptoms have been focused on the stage of AD close to dementia onset. Various disease progression and sub-type approaches have been proposed and developed. These include survival and multi-state models for investigating transitions between disease states (Hubbard and Zhou, 2011; Vos et al., 2013; van den Hout, 2016; Wei and Kryscio, 2016; Robitaille et al., 2018; Zhang et al., 2019); mixed effects models (linear, generalized, non-linear) that incorporate subject-specific random effects and can be extended to handle latent time shifts, random change points, latent factors, processes and classes, hidden states, and multiple outcomes (Hall et al., 2000; Jedynak et al., 2012; Liu et al., 2013; Proust-Lima et al., 2013; Donohue et al., 2014; Samtani et al., 2014; Lai et al., 2016; Zhang et al., 2016; Geifman et al., 2018; Li et al., 2018; Wang et al., 2018; Lorenzi et al., 2019; Proust-Lima et al., 2019; Villeneuve et al., 2019; Younes et al., 2019; Bachman et al., 2020; Kulason et al., 2020; Raket, 2020; Segalas et al., 2020; Williams et al., 2020) and can be combined with models for event-history data (Marioni et al., 2014; Blanche et al., 2015; Proust-Lima et al., 2016; Rouanet et al., 2016; Li et al., 2017; Iddi et al., 2019; Li and Luo, 2019; Wu et al., 2020); event-based models which attempt to model the pathological cascade of events occurring as the disease develops and progresses through disease stages (Fonteijn et al., 2012; Young et al., 2014; Chen et al., 2016; Goyal et al., 2018; Oxtoby et al., 2018); and various clustering approaches for discovering risk stratification/disease progression groups and endotypes. For example, those based on hierarchical, partitioning and model-based clustering algorithms/methods (Dong et al., 2016; Racine et al., 2016; Dong et al., 2017; ten Kate et al., 2018; Young et al., 2018). Moreover, various machine learning and other statistical approaches have been proposed for both disease progression, prediction and subgroup identification in Alzheimer’s disease (Fiot et al., 2014; Schmidt-Richberg et al., 2016; Cheng et al., 2017; Bhagwat et al., 2018; Khanna et al., 2018; de Jong et al., 2019; Martí-Juan et al., 2019; Brand et al., 2020; Golriz Khatami et al., 2020; Lei et al., 2020; Martí-Juan et al., 2020; Lin et al., 2021; Zhang et al., 2021).

However, in the earlier stages of disease, the development of disease models is far more challenging due to the relatively slow progression of the disease and clinical measures being insufficiently sensitive to detect such subtle changes. In order to develop disease models in the early stages when individuals do not have symptoms, or express only subjective complaints of cognitive decline or have only mild cognitive symptoms, it is necessary to undertake longitudinal follow-up of these individuals measuring reliable biomarkers of pathological changes alongside clinical outcomes. Ideally individuals would be followed-up over an extended period of time to ensure sufficient proportions make transitions through the various disease stages to dementia. Ultimately, these disease models would better inform patient selection into trials, improve understanding of AD progression in individuals and allow a more tailored approach to clinical management and targeting of disease modifying treatments to individuals (i.e., precision medicine) based on a range of biomarker modalities (e.g., neuroimaging, cerebrospinal fluid (CSF), blood), cognitive and clinical measures and risk factors.

Against this backdrop, the European Prevention of Alzheimer’s Dementia (EPAD) consortium (Ritchie et al., 2016) was initiated as a large public-private partnership, and funded by the Innovative Medicines Initiative (IMI) Joint Undertaking. A total of 39 European organisations or “partners” were involved in the EPAD consortium. EPAD was developed as an interdisciplinary research initiative with an aim of improving the understanding of the early stages of Alzheimer’s disease and delivering new preventative treatments.

The EPAD Longitudinal Cohort Study (LCS) was a prospective, multi-centre, pan-European study set up with the dual objectives of developing accurate longitudinal models over the entire course of Alzheimer’s disease (AD) prior to the onset of dementia and creating a trial-ready cohort for potential recruitment into the EPAD Proof-of-Concept (PoC) Trial (Solomon et al., 2018). It was designed as a long-term observational study with recruitment from different types of existing parent cohorts (PCs) across Europe (e.g., population-based, memory clinics) and then, later on, more directly from clinical settings. It aimed to provide both a well-phenotyped population covering the full continuum of risk of subsequent AD dementia development and enough participants with particular profiles potentially eligible for an adaptive designed trial. This aim was achieved through monitoring of the evolving characteristics of the EPAD cohort and use of a flexible and dynamic approach to selection into the LCS that allowed over- and under-sampling by particular characteristics already available in the PCs. The other component of the EPAD programme, the EPAD PoC Trial, was designed to provide an environment for testing multiple interventions for the secondary prevention of AD dementia.

Using the data collected in the LCS on cognitive and clinical outcomes, biomarkers and risk factors, we aim to develop state-of-the-art models for disease progression and stratification which can be used 1) to inform selective recruitment and adaptation in clinical trials, 2) for longitudinal prediction and stratification, 3) for subgroup identification based on both baseline and longitudinal biomarker profiles and, ultimately, 4) to help improve treatment and clinical management decisions. We adopt a two-stage approach, where we first identify subpopulations/classes with different underlying, potentially AD-related cognitive/functional trajectory patterns (i.e., latent clinical phenotypes) over time after controlling for known exogenous risk factors (constitutional and genetic). These latent phenotypes are then jointly modelled with endogenous neuroimaging and CSF biomarkers to identify homogeneous subgroups/clusters based on biomarker profiles (i.e., neuropathological endotypes) that are linked to these trajectory patterns.

## 2 Methods

### 2.1 Data

We performed all analyses on the V. IMI data release from the EPAD cohort (http://ep-ad.org/open-access-data/access/). Briefly, a total of 2,096 participants were screened and entered the cohort. Any participants who failed screening, had a baseline global clinical dementia rating (CDR) ≥1, or had a diagnosis of Alzheimer’s dementia at baseline were excluded, leaving 1,828 eligible participants. Participants were aged at least 50 years old, with either a CDR global score of 0 (*n* = 1,313) or 0.5 (*n* = 498) (The CDR global scores for seventeen participants were missing.) Recruitment occurred across 31 centres from 10 different European countries. Follow-up visits were designed to occur at 6 months, 1 year and yearly thereafter. Unfortunately, the LCS closed at the end of the IMI-funding period and therefore the maximum number of visits was five. Of the 1,828 participants with a baseline visit, 1,567 attended the 6-months visit, 1,188 attended the 1-year visit and 396 and 89 attended the 2-years and 3-years visits respectively. Two hundred and fifty four participants only had a baseline visit, 389 had two visits (including five who had a baseline and 1-year visit but not 6-months), 791 had three visits (including 2 who had baseline, 1-year and 2-years visits but not a 6-months visit; the remaining attended the first three visits), 307 had four visits (including 2 who had baseline, 6-months, 2-years and 3-years visits but not a 1-year visit; the remaining had all visits up to 2 years) and 87 had five visits. We restrict our study to the 1,574 participants who had more than one visit.

The variables used in the models can be considered to belong to four domains: 1) outcomes, 2) baseline risk factors, 3) baseline biomarkers, and 4) longitudinal biomarkers.

#### Outcomes

The outcomes used were transformations of CDR sum of boxes (CDRSB) and Mini-Mental State Examination (MMSE) scores. To deal with floor and ceiling effects of CDRSB, a logistic transformation was applied to CDRSB as defined in Eq. 1:

$$tCDRSB=-\log\left(\frac{\left(CDRSB+0.1\right)}{\left(18-CDRSB+0.1\right)}\right)$$ (1)

A normalising transformation was applied to MMSE values, converting MMSE from a 0–30 scale to nMMSE on a 0–100 scale to deal with curvilinearity (Philipps et al., 2014). CDRSB was scheduled to be collected at all visits but MMSE was not designed to be collected at the 6-months visit.

#### Baseline Risk Factors

Baseline risk factors included age, sex, education, family history of AD (first-degree relatives), and APOE*ϵ*4 carrier status. Age is treated as a continuous variable. Sex, family history, and APOE are binary. Education was recorded in the LCS as years of formal education. However, as the values have different interpretations for different countries, years of education was converted to a three-category highest educational attainment level variable labelled 1, 2, and 3 on a country-specific basis (European Commission/EACEA/Eurydice, 2018). Level 1 is defined as up to secondary education, level 2 as beyond secondary education up to undergraduate ordinary degree, and level 3 as postgraduate studies.

#### Baseline Biomarkers

Baseline biomarkers included:

• the ratio of phosphorylated tau (pTau) to amyloid-beta 42 (A*β*), derived from CSF samples using the fully automated Roche Elecsys System in a single laboratory;
• volumetric imaging variables of the total of the left and right hippocampi and of the total of the four ventricles adjusting for head size by dividing by the pseudo total intracranial factor (HV and VV), processed by IXICO using the learning embeddings for atlas propagation (LEAP) method (Wolz et al., 2010);
• neurological radiological reads variables obtained through central assessment of magnetic resonance (MR) images by IXICO raters following a standardised, compliant and efficient workflow (Ritchie et al., 2020; ten Kate et al., 2018):
– average of left and right medial temporal lobe atrophy (MTA);
– Fazekas scale deep (FSD) and Fazekas scale periventricular (FSPV); and
– five regional age-related white matter change (ARWMC) variables.

For EPAD participants, values of pTau/A*β* > 0.024 are here defined as CSF “AD positive” based on the biomarker cut-offs derived by Roche for EPAD using the methodology in (Hansson et al., 2018; Schindler et al., 2018), and reflect either decreased concentrations of A*β* (a marker of amyloidosis) or increased levels of pTau (a marker of neurofibrillary tangles). All radiological reads biomarkers were converted to binary variables <1 and ≥1, except for Fazekas scale deep which was dichotomised instead at 2. A score of 0 for all radiological reads variables indicates no pathology and scores ≥1 (and ≥2 for Fazekas scale deep) indicate some pathology. A score of 0.5 in the average of left and right MTA is assumed to provide inconclusive evidence of pathology. A combined ARWMC variable was created that counted the number of age-related regions with evidence of white matter lesion cerebrovascular pathology, and a count ≥3 indicated that the majority of regions had signs of pathology.

#### Longitudinal Biomarkers

Longitudinal biomarkers considered were derived from the MR volumetric imaging variables of total hippocampal volume and total ventricular volume adjusting for head size. The processing of the longitudinal volumetric variables was also performed by IXICO using LEAP. The rates of change in the adjusted total hippocampal and ventricles volumes were calculated by dividing the difference between the last observed and baseline volumes by the time in study (in years) between the taking of the last and baseline volumes. These rate of change (i.e., annualised change) variables were used in our analyses to describe the longitudinal changes in biomarkers.

### 2.2 Statistical Methods

**FIGURE 1:**
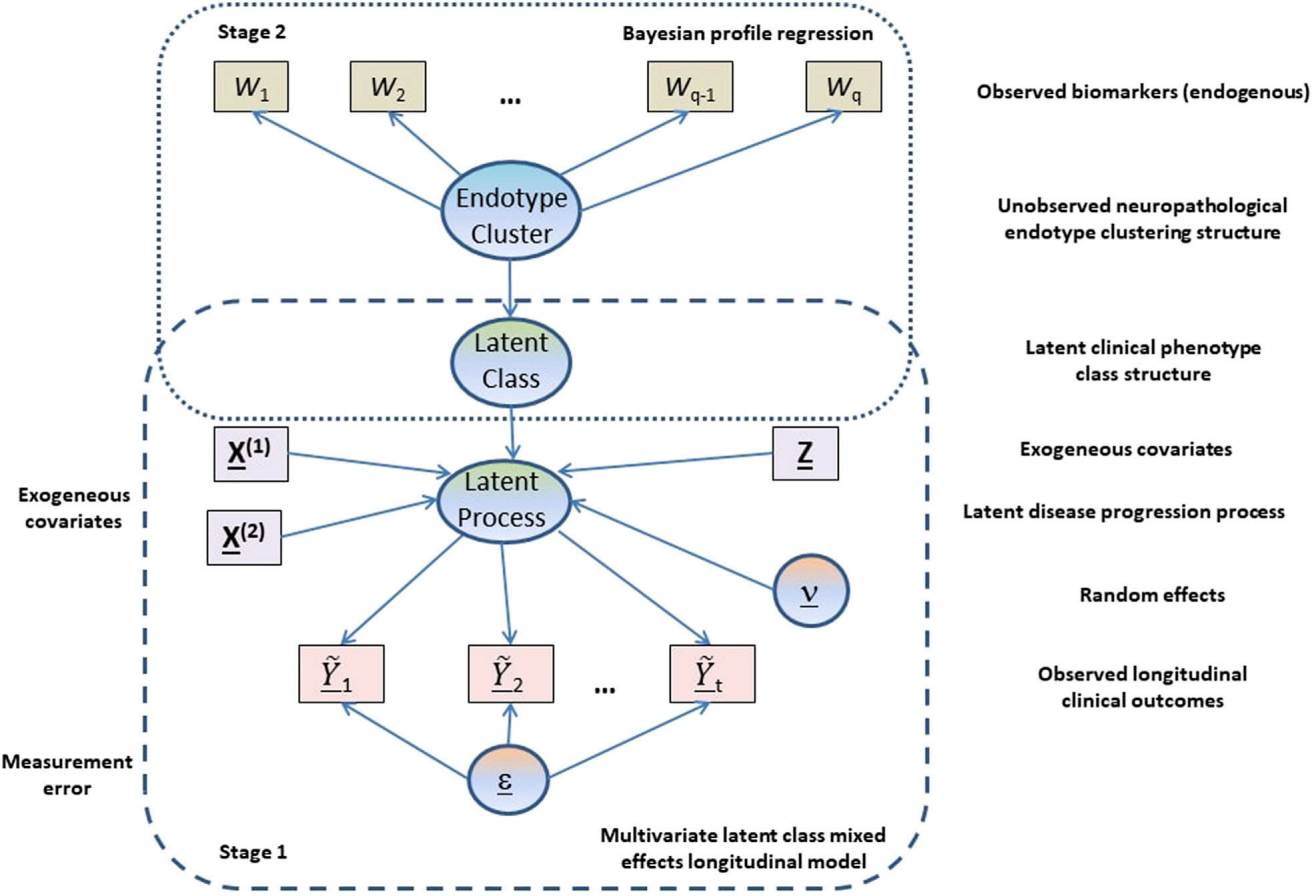
Graphical representation of the proposed two-stage approach.

Our analysis is based on a two-stage approach (see Figure 1) where in the first stage a multivariate latent class linear mixed effects modelling approach is adopted to model the longitudinal cognitive and clinical outcomes adjusting for constitutional and genetic risk factors purported to be important in AD disease progression or related to selection into the EPAD LCS. From the multivariate latent class linear mixed effects model, latent clinical phenotypes corresponding to the latent classes are extracted to characterise the various mean trajectory profiles which individuals may follow over time. These latent phenotypes result from a hard assignment of individuals to specific latent classes based on their posterior probabilities of class membership. In the second stage, a probabilistic outcome-guided clustering approach based on Dirichlet process mixture modelling called Bayesian profile regression is applied to the latent phenotypes alongside the CSF and neuroimaging biomarkers. This aims to identify homogeneous clusters of participants with particular neuropathological endotypes characterised by biomarker profiles linked to clinical disease progression. Note that the latent phenotypes and endotypes are not meant to represent a grouping orthogonal to disease severity or stage, but reflect and characterise potential underlying processes and features that give rise to or are associated with disease severity or stage.

The specific statistical formulation of this two-stage modelling approach for disease progression, trajectory stratification and subgroup identification are outlined in the next two subsections. Missing response data are assumed to be missing at random (MAR) for both stages, which allows valid inference using likelihood approaches.

#### 2.2.1 Multivariate Latent Class Linear Mixed Effects Model

We used a multivariate latent class linear mixed effects model (MLCMM) to identify *G* mean profiles of trajectories corresponding to *G* latent classes or sub-populations of individuals (Lai et al., 2016; Proust-Lima et al., 2017). This model assumes that a latent process Λ_*i*_(*t*) generates the *K* longitudinal outcomes at time *t*, and this latent process is characterised by the mean trajectory profile corresponding to the latent class membership of individual *i*. *Y*_*ijk*_ is a measure of outcome *k* (*k* = 1, …, *K*) for subject *i* (*i* = 1, …, *N*) at measurement occasion *j* (*j* = 1, …, *n*_*ik*_), with associated time of outcome measurement from start of study *t*_*ijk*_. *Y*_*ijk*_ is related to Λ_*i*_ (*t*_*ijk*_) via an outcome-specific link function. Here for the purposes of this paper, we assume a linear transformation link function (others are possible) for the outcomes with outcome-specific parameters. That is, ${Y\sim }_{ijk}=\frac{Y_{ijk}-\eta_{1k}}{\eta_{2k}}$, *k* = 1, …, *K*. These transformations allow the transformed outcomes to be interpreted as noisy measurements of the underlying latent process with outcome-specific measurement errors.

The general formulation of the linear mixed effects part of our model given membership to latent class *g* is

$${\tilde{Y}}_{ijk}{|}_{c_{i}=g}=\Lambda_{i}\left(t_{ijk}\right){|}_{c_{i}=g}+\epsilon_{ijk}$$ (2)

with

$$\Lambda_{i}\left(t_{ijk}\right){|}_{c_{i}=g}=X_{ijk}^{\left(1\right)T}\beta +X_{ijk}^{\left(2\right)T}\gamma_{g}+Z_{ijk}^{T}\upsilon_{ig},$$ (3)

where *c*_*i*_ is the latent class variable, $X_{ijk}^{\left(1\right)}$ are the covariates associated with the class-independent fixed effects *β* and $X_{ijk}^{\left(2\right)}$ are the covariates associated with the class-specific fixed effects *γ*_*g*_. *Z*_*ijk*_ are the covariates associated with the class-specific random effects *υ*_*ig*_, which are from a zero-mean multivariate normal with variance-covariance matrix *ω*_*g*_ *B*, where *B* is left unspecified and *ω*_*g*_ is a positive proportionality factor (with *ω*_*G*_ = 1 to ensure identifiability). The measurement errors {*ϵ*_*ijk*_} are assumed to be independent Gaussian random variables with mean 0 and outcome-specific variances $\sigma_{k}^{2}\;\left(k=1,\ldots ,K\right)$.

The latent variable *c*_*i*_ equals *g* when subject *i* belongs to latent class *g*. To complete the specification of our multivariate latent class mixed model, the probability of individual *i* belonging to class *g* is described by the multinomial logistic submodel without covariates given by Eq. 4:

$$\pi_{ig}=P\left(c_{i}=g\right)=\frac{e^{\xi_{0g}}}{\sum_{l=1}^{G}e^{\xi_{0l}}},$$ (4)

where *ξ*_0*g*_ is the intercept parameter for class *g*. Extension of this latent class membership submodel to include covariates is straightforward. The full MLCMM is fitted using maximum likelihood estimation within R (R Core Team, 2017) using the multlcmm function in the lcmm package (Proust-Lima et al., 2017).

In our application, we included the logistic transformed CDRSB, tCDRSB, and normalised MMSE, nMMSE, as outcomes (*K* = 2) in our MLCMM formulation. For both these outcomes and the latent process, a higher value indicates less cognitive/functional impairment (i.e., better cognitive functioning). We used time in study as the time scale and allowed class-specific fixed intercepts and slopes (time in study effects). As maximum follow-up in the EPAD study population was 3 years and 4 months and the majority of subjects had two or three visits, we considered only linear trends in an individual’s underlying disease process. The baseline risk factors described in Section 2.1 were introduced into Eq. 3 with associated class-independent fixed effects. We included only class-specific random intercepts into the latent process model, which are introduced to induce correlation across the longitudinal observations of an outcome for an individual and to better align participants in terms of where they fall on the disease time scale. The variance of the random intercept for the reference class is not estimated by the model and is set to be 1. The best choice of the number of latent classes was made using the Bayesian Information Criterion (BIC) and the relative entropy.

All observations with either a recorded CDRSB or MMSE were considered for inclusion in the model provided that individuals had 2 or more visits. These corresponded to 4,795 visits on 1,574 participants. Of which, there were 3,228 visits with both CDRSB and MMSE present, 1,558 visits with only CDRSB present, and nine visits with only MMSE present. Of the 1,574 individuals, 86 had five observation-visits, 305, 789, 384 and 10 had 4, 3, 2 and 1 observation-visits with either CDRSB or MMSE or both present respectively. However, 31 individuals had missing APOE*ϵ*4 carrier status information and were excluded. This thus resulted in 1,543 individuals be included in the MLCMM analysis.

#### 2.2.2 Bayesian Profile Regression

Bayesian profile regression (Molitor et al., 2010) is a non-parametric outcome-guided clustering approach that links an outcome variable to covariates via cluster membership. Here, it was applied to identify G* clusters of participants, with each cluster characterised by particular clinical disease progression phenotypes (latent classes from the MLCMM analysis) and a particular CSF/neuroimaging biomarker profile. These clusters can be interepreted as corresponding to different neuropathological endotypes.

Bayesian profile regression uses a Dirichlet process mixture model (DPMM), which can be regarded as the limit of a finite mixture model as the number of components goes to infinity. That is, for observed data *D*_*i*_ for subject *i*, we have the following DPMM likelihood:

$$\begin{array}{ll} p\left(D_{i}|\pi *,\Theta \right) & =\overset{\infty }{\underset{h=1}{\sum }}p\left({c*}_{i}=h|\pi *\right)p\left(D_{i}|{c*}_{i}=h,\Theta \right) \end{array}$$ (5)

$$\begin{array}{l} =\overset{\infty }{\underset{h=1}{\sum }}{\pi *}_{h}f\left(D_{i}|\Theta_{h}\right), \end{array}$$ (6)

where ${c*}_{i}\in Z^{+}$ denotes latent cluster membership, $\pi *={\left({\pi *}_{1},{\pi *}_{2},\ldots \right)}^{T}$ are mixture component (cluster) weights and $\Theta^{T}=\left(\Theta_{1}^{T},\Theta_{2}^{T},\ldots \right)$ are component-specific parameters for the mixture component densities, indexed by $h\in Z^{+}$.

In addition to covariates *W*_*i*_ for subject *i*, Bayesian profile regression models an outcome $Y_{i}^{*}$ that also informs the clustering and is assumed to be conditionally independent of the covariates given cluster assignment $c_{i}^{*}$. Furthermore, covariates can be a mix of discrete and continuous, $W_{i}^{T}=\left(W_{i}^{\left(d\right)T},W_{i}^{\left(c\right)T}\right)$, with discrete covariates $W_{i}^{\left(d\right)}$ and continuous covariates $W_{i}^{\left(c\right)}$ also assumed to be conditionally independent given $c_{i}^{*}$. We therefore have observed data $D_{i}^{T}=\left(Y_{i}^{*},W_{i}^{\left(d\right)T},W_{i}^{\left(c\right)T}\right)$ for subject *i* and Eq. 6 becomes

$$p\left(Y_{i}^{*},W_{i}^{\left(d\right)},W_{i}^{\left(c\right)}|\pi *,\Theta \right)=\overset{\infty }{\underset{h=1}{\sum }}{\pi *}_{h}f\left(Y_{i}^{*}|\Theta_{h}^{\left(o\right)}\right)f\left(W_{i}^{\left(d\right)}|\Theta_{h}^{\left(d\right)}\right)f\left(W_{i}^{\left(c\right)}|\Theta_{h}^{\left(c\right)}\right),$$ (7)

where $\Theta_{h}^{T}=\left(\Theta_{h}^{\left(o\right)T},\Theta_{h}^{\left(d\right)T},\Theta_{h}^{\left(c\right)T}\right)$ are the component-specific parameters for the outcome, discrete covariate and continuous covariate densities respectively.

The stick-breaking construction of the DPMM (Sethuraman, 1994) is used within Bayesian profile regression which gives the following formulation for the prior on the mixture weights: ${\pi *}_{1}=V_{1}$ and ${\pi *}_{h}=V_{h}\prod_{l<h}\left(1-V_{l}\right)$ for *h* ≥ 2 with $V_{h}\overset{iid}{∼}\text{Beta}\left(1,\alpha \right)$. The concentration hyperparameter *α*, which itself has a gamma prior distribution, affects the mixture weight distribution and implicitly informs the number of non-empty clusters. One of the key desirable properties of a DPMM approach to clustering is the removal of the need to pre-specify the number of clusters. Prior distributions are also placed on the component-specific parameters Θ_*h*_ and Markov chain Monte Carlo (MCMC) is used to fit the resulting profile regression model (see (Liverani et al., 2015) for details of the prior distributions and for computational aspects of the MCMC).

In our application, the outcome variable for each subject is the latent class predicted from the MLCMM analysis, i.e. Yi*=c^i. This is treated as a categorical variable with cluster-dependent parameters: $Y_{i}^{*}|\Theta_{h}^{\left(o\right)}∼Cat\;\left(\theta_{h,1}^{\left(o\right)},\theta_{h,2}^{\left(o\right)},\ldots ,\theta_{h,\hat{G}}^{\left(o\right)}\right)$, where G^ is the estimated number of latent classes in the MLCMM. The covariates used in the model are the baseline and longitudinal biomarkers described in Section 2.1. In particular, we included five binary baseline covariates (pTau/A*β*, MTA, FSD, FSPV, ARWMC combined) for each subject, each independently taking a Bernoulli distribution given cluster assigmment: $W_{i,q}^{\left(d\right)}|\Theta_{h,q}^{\left(d\right)}∼Bern\;\left(\theta_{h,q}^{\left(d\right)}\right)$ for *q* = 1, …, 5. Additionally, four continuous covariates (standardised) were included—adjusted total hippocampal and ventricles volumes at baseline, HV and VV, and their corresponding longitudinal rate of changes, HV rate and VV rate—jointly taking a multivariate Gaussian distribution given cluster assignment: $W_{i}^{\left(c\right)}|\Theta_{h}^{\left(c\right)}∼N_{4}\;\left(\mu_{h},\Sigma_{h}\right)$. This allows for the correlation between the continuous covariates to be taken into account.

Since the clustering assignments and number of clusters vary across the MCMC iterations, it is useful to obtain a “representative” clustering that summarises the MCMC output. Following (Molitor et al., 2010; Liverani et al., 2015), we find a “representative” clustering based on the *N* × *N* posterior similarity matrix *S*, where element (*i*, *j*) of *S* is the proportion of MCMC iterations where subjects *i* and *j* are assigned to the same cluster. The partitioning around medoids (PAM) clustering algorithm (Kaufman and Rousseeuw, 1990) is applied to the posterior dissimilarity matrix 1 − *S* to find a clustering of the subjects that is consistent with *S*, with the optimal number of clusters selected using the silhouette width method (Rousseeuw, 1987).

An advantage of the DPMM clustering framework is that it takes uncertainty in the clustering (including the number of clusters) into account. This allows the uncertainty associated with the “representative” clustering to be investigated. If we let $C_{h}^{\left(rep\right)}$ denote the subset of subjects allocated to cluster *h* in the “representative” clustering, then at MCMC iteration *r* we can calculate the average value of mixture component parameters for subjects in $C_{h}^{\left(rep\right)}$. For example, for the Bernoulli distribution parameter for binary covariate *q* we calculate

$${\bar{\theta }}_{h,q}^{\left(d\right)}\left(r\right)=\frac{1}{n_{h}}\underset{i\in C_{h}^{\left(rep\right)}}{\sum }\theta_{c_{i}^{*}\left(r\right),q}^{\left(d\right)}\left(r\right)$$ (8)

where *n*_*h*_ is the number of subjects in $C_{h}^{\left(rep\right)}$ and $\theta_{c_{i}^{*}\left(r\right),q}^{\left(d\right)}\left(r\right)$ is the sampled Bernoulii parameter for the cluster $c_{i}^{*}\left(r\right)$ that subject *i* is allocated to at MCMC iteration *r*. The distribution of ${\bar{\theta }}_{h,q}^{\left(d\right)}\left(r\right)$ across the MCMC iterations (i.e., the posterior distribution) gives an insight into the uncertainty of cluster *h* in the “representative” clustering; narrower credible intervals indicates a more consistent clustering. These distributions can be computed for all of the “representative” clusters and for all of the mixture component parameters associated with the outcome variable and covariates.

Bayesian profile regression is implemented in the R package PReMiuM (Liverani et al., 2015) and this was used to fit the model and perform the post-processing analysis (PReMiuM package version 3.2.3; R version 3.6.3; default settings for hyperparameters used; run for 350,000 MCMC iterations with first 100,000 discarded as burn-in). Convergence of the MCMC procedure was investigated by checking agreement between the “representative” clusterings from six independent chains (quantified using the adjusted Rand index) and by inspection of posterior parameters (see (Liverani et al., 2015) for more details of convergence diagnostics). Consensus clustering of the consensus dissimilarity matrix, obtained through averaging of the dissimilarity matrices from the six independent chains and applying PAM to this matrix, resulted in the final representative clustering structure. The adjusted Rand indices assessing agreement between the final representative consensus clustering with the representative clusterings from the six independent chains are calculated and reported. Moreover, the lower triangular part of the individual posterior dissimilarity matrices from the six independent chains are compared to the lower triangular part of the consensus posterior dissimilarity matrix using Pearson’s correlation. Risk and covariate profiles are derived through pooling of MCMC iterations across the six chains and using the final representative consensus clustering. Additionally, Bayesian profile regression without the latent classes as outcome was performed to obtain a baseline/reference clustering structure based purely on the biomarkers. All 1,543 subjects included in the MLCMM analysis were included in the Bayesian profile regression analysis.

#### 2.2.3 Validation

The final results of our multivariate latent class mixed model and Bayesian profile regression analysis on the full data-set were assessed for class and cluster validity through stability assessment under repeated sub-setting. We repeatedly (i.e., ten times) split the full data-set into two subsets, by first stratifying the full data-set by number of visits and then randomly allocating (with equal probability) within each strata a participant to belong to either the first or second subset. Our proposed two-stage approach is then applied in turn to each subset to estimate a latent class structure followed by clustering structure as described in the previous two subsections. For assessing class validity for each split, we begin by using the multivariate latent class model trained on one subset to predict the class membership of participants in the other subset and vice versa. We next cross-tabulate the out-of-sample predictions of class memberships (based on the model trained on the subset that does not include the participant for whom a prediction is being made) with the in-sample class membership assignments (obtained from the model trained on the subset which includes the participant for whom a prediction is being made) to assess out-of-sample performances in the models trained on the two subsets and stability of class structure across the two subsets. Cohen’s kappa statistic and the adjusted Rand index are used to measure the out-of-sample performances and across subset stability. Finally, the validity/stability of the class structure obtained from the full data-set is evaluated by a comparison of the in-sample class assignments based on the final multivariate latent class mixed model on the full data-set to the in-sample class assignments obtained from the multivariate latent class models for the subsets, using again Cohen’s kappa and the adjusted Rand index.

For assessing clustering validity, we first apply Bayesian profile regression to the biomarker and latent class assignment data for each subset in turn and obtain the consensus results over six chains as described earlier. Next, the consensus dissimilarity matrices for the subsets are compared to their corresponding block diagonals of the consensus dissimilarity matrix from our Bayesian profile regression on the full data-set using Pearson’s correlation. To assess cluster stability, the corresponding PAM consensus representative clustering structures from each subset are compared to the final representative clustering from the full data-set using the adjusted Rand index. Moreover, we make predictions for the held-out subsets that allow us to compare 1) their predicted dissimilarity matrices with the corresponding off-diagonal blocks of the final consensus dissimilarity matrix from the full data-set using Pearson’s correlation, and 2) their predicted clustering structures with the PAM consensus representative clustering obtained using a model trained on the held-out subset (clustering predictions are obtained by using the predicted dissimilarity matrices to assign participants in the held-out subset to the PAM consensus representative cluster from the training subset that they are closest to).

External validation was not possible as we do not have access to data from studies on similar populations with the corresponding extensive baseline and longitudinal biomarker and phenotypic information to EPAD.

## 3 Results

### 3.1 Baseline Characteristics of the European Prevention of Alzheimer’s Dementia Longitudinal Cohort Study Population

Table 1 describes the group of 1,574 participants with two or more visits in the EPAD longitudinal cohort. The mean age of these participants was 65.4 years with a standard deviation of 7.4 years. Around 56% were female and 63% had their highest educational attainment beyond secondary education—an indication of a highly educated cohort of participants recruited; reflecting the eligibility criterion on minimum years of formal education. The cohort was enriched for participants with a family history of AD (first degree relatives) and APOE*ϵ*4 carriers, without diminished decision-making capacity. For the group, this enrichment corresponded to 65.5 and 37.5% with a known family history of AD and a known carrier for APOE*ϵ*4 respectively. 78% of this group (*n* = 1,226) had a global CDR of 0, while the remaining 22% had a score of 0.5 (*n* = 346); two participants had unknown baseline CDR global. Around 82% of those with a family history of AD had a CDR global score of 0. Whereas 70% of those without a family history of AD had a CDR score of 0. Thus there was a clear association between CDR global score and family history of AD favouring the recruitment of participants with a family history who do not have any baseline cognitive impairment and for those without a family history enriching for early symptomatics (*p* < 0.0001; *χ* ^2^-test). No evidence for an association between CDR global score and APOE*ϵ*4 carrier status was found (*p* = 0.10), with 80% of non-carriers and 76% of carriers having CDR global equal 0.

**TABLE 1:** Baseline characteristics of the 1,574 participants with more than one visit.

	Variable		Mean (SD)	Frequency (%)	No. Unknown
Risk Factors	Age, years		65.4 (7.4)		0
	Sex	Female		888 (56.4)	0
		Male		686 (43.6)	
	Education	Level 1		587 (37.3)	0
		Level 2		393 (25.0)	
		Level 3		594 (37.7)	
	Family history of AD	No		543 (34.5)	0
		Yes		1,031 (65.5)	
	APOE*ϵ*4 carrier	No		965 (62.5)	31
		Yes		578 (37.5)	
Outcomes	CDRSB	0		1,162 (73.9)	2
		0.5		214 (13.6)	
		≥1		196 (12.5)	
	MMSE	29–30		999 (63.5)	1
		27–28		417 (26.5)	
		≤26		157 (10.0)	
	Transformed CDRSB, tCDRSB		4.60 (1.04)		2
	Normalised MMSE, nMMSE		83.6 (14.6)		1
Biomarkers	pTau/A*β*	≤0.024		1,240 (80.5)	33
		>0.024		301 (19.5)	
	MTA average	0		800 (51.2)	13
		0.5		375 (24.0)	
		≥1		386 (24.7)	
	Fazekas scale deep	<2		1,317 (84.4)	13
		≥2		244 (15.6)	
	Fazekas scale periventricular	<1		947 (60.7)	13
		≥1		614 (39.3)	
	ARWMC basal ganglia	<1		1,379 (88.3)	13
		≥1		182 (11.7)	
	ARWMC frontal	<1		506 (32.4)	13
		≥1		1,055 (67.6)	
	ARWMC infratentorial	<1		1,465 (93.9)	13
		≥1		96 (6.1)	
	ARWMC parieto-occipital	<1		786 (50.4)	13
		≥1		775 (49.6)	
	ARWMC temporal	<1		1,268 (81.2)	13
		≥1		293 (18.8)	
	ARWMC combined	<3		1,194 (76.5)	13
		≥3		367 (23.5)	
	Total hippocampal volume (adj), *mm* ^3^		5,793 (703)		62
	Total ventricular volume (adj), *mm* ^3^		32,991 (17,669)		168

Table 1 also summarises the distributions of the EPAD cognitive and clinical outcomes and CSF and neuroimaging biomarkers at baseline. Ten percent of participants had an MMSE score below 27 and 12.5% had a CDRSB score of 1 or above; suggesting that the majority of participants had high levels of cognitive functioning at baseline. However, varying degrees of disease pathology at baseline were indicated on considering a range of biomarkers. AD positivity was estimated around 20% using the ratio of phosphorylated tau to amyloid-beta 42 in CSF. Convincing evidence for the widening of the choroid fissure to different degrees (average of left and right MTA ≥1) was found in about a quarter of the participants, whilst varying percentages of white matter lesion cerebrovascular pathology were seen ranging from 6 to 68% based on age-related regional white matter changes or based on an overall impression of the brain using the Fazekas scales (approximately 16 and 39%). Nearly a quarter of the participants (23.5%) had indications of cerebrovascular pathology in three or more of the five age-related white matter regions. The mean adjusted total hippocampal and ventricles volumes at baseline (with standard deviation) were 5,793mm^3^ (703mm^3^) and 32,991mm^3^ (17,669mm^3^).

### 3.2 Disease Progression and Latent Phenotypes—Results From MLCMM

**FIGURE 2:**
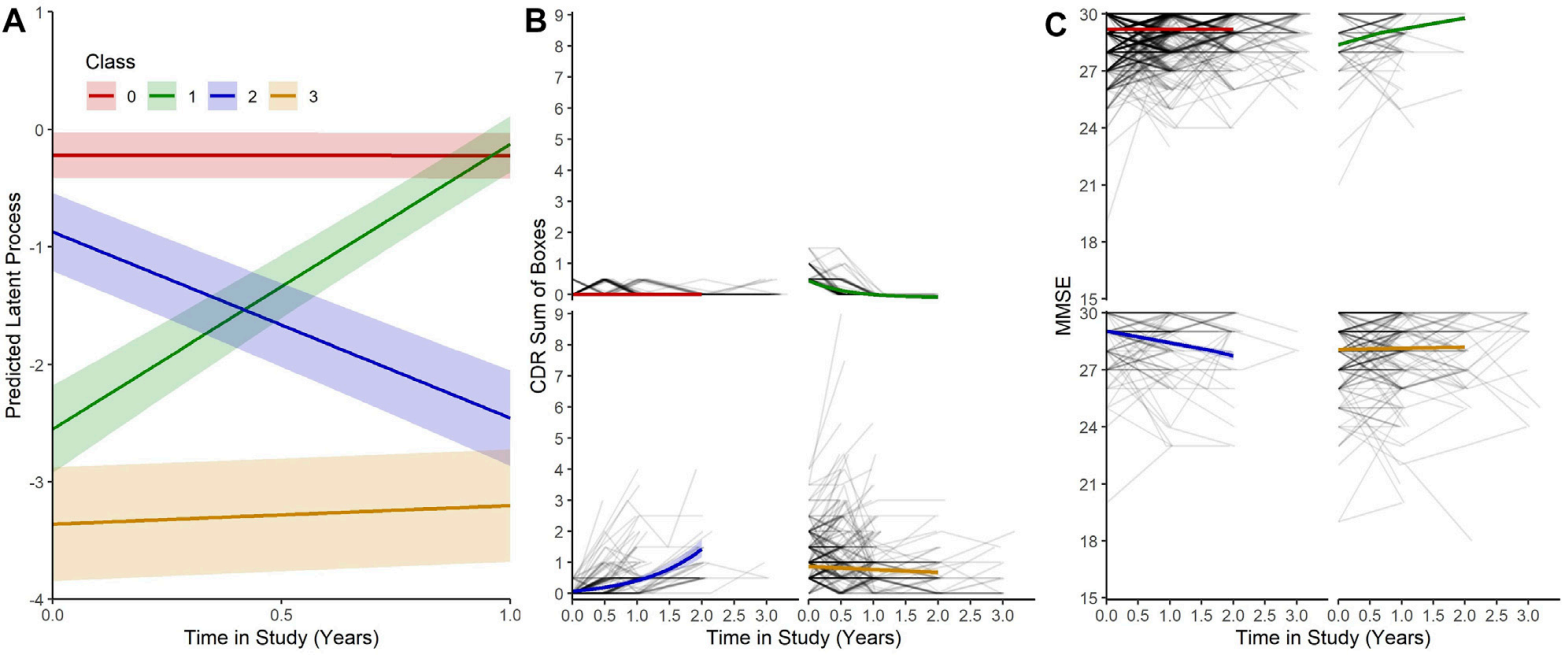
**(A)** Predicted trajectories with 95% confidence bands for each class on the latent process scale given mean values for each of the covariates. **(B)** and **(C)** Predicted trajectories with 95% confidence bands for each class on the CDRSB and MMSE scale given mean values for the each of the covariates with observed outcomes for each participant.

Our MLCMM was able to identify four distinct mean trajectories. Figure 2 shows these four mean trajectory profiles on the latent process scale and on the original scales for CDRSB and MMSE. Latent clinical phenotype classes 0 to 3 had, respectively, 1,050 (68.0%), 97 (6.3%), 106 (6.9%), and 290 (18.8%) individuals hard assigned to them based on a posterior classification of participants’ class membership through the selection of the participant’s class with the highest posterior class-membership probability. Latent phenotype class 0, which had the majority of participants, is characterised by individuals having the highest levels of cognitive functioning with no signs of impairment at baseline and no decline throughout the course of the study. Class 1 contained individuals who showed some signs of cognitive/functional impairment at baseline but appeared to improve over time. Class 2 was characterised by individuals who appeared cognitively and functionally unimpaired at baseline (although cognitive functioning levels were not as high as those in class 0) but then declined on follow-up. Whereas class 3 contained individuals who showed the most evident signs of early cognitive/functional impairment at baseline and continued to show impairment on follow-up.

Table 2 reports the results from our four-class MLCMM. A higher baseline age and a lower level of education are associated with higher levels of cognitive/functional impairment; consistent with findings from the neurodegenerative and AD literature. Due to how individuals were recruited into the study (through use of a flexible and dynamic approach to selection), biased effects of family history of AD and APOE*ϵ*4 carrier status were expected and therefore the corresponding estimates of these effects were not interpreted as they were notably affected by the selection mechanism. For example, both were found not to be statistically significantly associated with cognitive/functional impairment and the effect of family history of AD was in the opposite direction to that reported in the literature.

**TABLE 2:** Results of the 4-class MLCMM on the 1,543 participants.

		Coefficient (SE)	*p*-value
Class membership model	Intercept class 0	1.30 (0.07)	<0.0001
	Intercept class 1	−1.04 (0.12)	<0.0001
	Intercept class 2	−0.87 (0.13)	<0.0001
Fixed effects model	Intercept class 0	0 (not estimated)	—
	Intercept class 1	−2.33 (0.15)	<0.0001
	Intercept class 2	−0.65 (0.14)	<0.0001
	Intercept class 3	−3.14 (0.22)	<0.0001
	Time in study class 0	−0.0040 (0.014)	0.773
	Time in study class 1	2.43 (0.16)	<0.0001
	Time in study class 2	−1.58 (0.11)	<0.0001
	Time in study class 3	0.16 (0.04)	0.0001
	Age	−0.0033 (0.0014)	0.022
	Sex male	−0.015 (0.019)	0.443
	Education level 2	0.022 (0.024)	0.367
	Education level 3	0.043 (0.022)	0.049
	Family history of AD	0.016 (0.021)	0.453
	APOE*ϵ*4	−0.021 (0.019)	0.290
Link function parameters	tCDRSB *η*_1_	5.31 (0.07)	<0.0001
	tCDRSB *η*_2_	0.73 (0.04)	<0.0001
	nMMSE *η*_1_	87.96 (0.48)	<0.0001
	nMMSE *η*_2_	3.80 (0.30)	<0.0001

The measurement error variances for tCDRSB and nMMSE are 0.531 and 3.527 respectively indicating that tCDRSB has a stronger relationship to the underlying latent disease process. The estimated class-specific proportionality factors, ${\hat{\omega }}_{g}\;\left(g=0,1,2\right)$, which here correspond to the variances of the class-specific random intercepts are 0.0004 for class 0, 0.397 for class 1, and 0.957 for class 2. (The variance for the random intercept corresponding to class 3 was set to 1 for identifiability.) The log-likelihood of this model was −16,842.95, the BIC 33,869.44, and the (relative) entropy 0.947. By comparison, the equivalent three-class model had a log-likelihood of −17,097.26, a higher BIC of 34,348.70, and a lower entropy of 0.932. Thus our four-class model was preferred. It showed excellent ability to discriminate between latent trajectory classes.

Moreover assessment of validity of our four-class model through class stability under repeated sub-setting gave mean Cohen’s kappa and adjusted Rand index values (with standard deviations) of 0.987 (0.008) and 0.989 (0.006), respectively, across the twenty subset comparisons and 0.993 (0.004) and 0.995 (0.003) for the ten comparisons against the full data-set. These results indicate near perfect agreement with evidence for stability across subsets and validity of the class structure derived based on the full data-set. Across the ten splits, the number of discordant classifications seen when the in-sample latent class membership predictions for subsets are compared to the class memberships predicted by our four-class model on the full data-set ranged from 3 to 13 out of the 1,543 participants (0.19–0.84%). For the twenty subsets across the ten splits, four-class multivariate latent class mixed models were always found to provide a better fit (based on BIC) than the alternative three-class multivariate latent class mixed models, and these four-class models had similar class structure as our four-class model on the full data-set.

We further characterised these four latent phenotype classes by baseline and change variables and (marginally) compared these variables across classes using analysis of variance (ANOVA) tests for the continuous variables and *χ* ^2^ tests for binary and categorical variables. The results are shown in Table 3. We observe increasing trends in mean age and mean baseline ventricles volume across the latent classes from 0 to 3 and a decreasing trend in mean baseline hippocampal volume. Class 3 differed from the other three classes in having the highest proportions of males, lowest educational level attainers, those with AD positivity at baseline and with evidence on baseline MTA of widening of choroid fissure in varying degrees from widen to end stage atrophy. There was evidence found for differences amongst the classes in the presence of white matter hyperintensities in the entire brain as measured by the Fazekas scales, with latent class 2 having the highest proportion of participants with abnormal pathology. No evidence of any further differences between classes 3 and 2 (or between classes 0 and 1) was found with regard to age-related regional white matter changes. However evidence of differences between the lower two classes (0 and 1) compared to the upper two classes (2 and 3) was found for a number of these neuroimaging variables associated with white matter lesions (Table 3).

**TABLE 3:** Characterisation of the baseline and change variables by latent phenotype classes.

Mean (SD)						
Variable		Class 0	Class 1	Class 2	Class 3	ANOVA *p*-value
Age, years		63.9 (7.0)	65.6 (6.6)	68.1 (7.0)	69.5 (7.0)	<0.0001
Total hippocampal						
volume (adj), *mm* ^3^		5,911 (644)	5,814 (725)	5,609 (715)	5,429 (768)	<0.0001
Total ventricular						
volume (adj), *mm* ^3^		30,715 (16,348)	35,404 (19,823)	37,962 (18,838)	38,396 (19,405)	<0.0001
Annual (adj) hippocampal						
volume change, *mm* ^3^/*yr*		−9.4 (83.5)	−30.4 (61.9)	−40.2 (85.3)	−55.3 (99.1)	<0.0001
Annual (adj) ventricular						
volume change, *mm* ^3^/*yr*		988 (910)	1,430 (1,354)	1,958 (1,651)	1,688 (1,586)	<0.0001

Frequency (%)						
Variable		Class 0	Class 1	Class 2	Class 3	*χ*^2^*p*-value
Sex	Female	614 (58.5)	54 (55.7)	65 (61.3)	137 (47.2)	0.005
	Male	436 (41.5)	43 (44.3)	41 (38.7)	153 (52.8)	—
Education	Level 1	368 (35.0)	35 (36.1)	34 (32.1)	138 (47.6)	0.009
	Level 2	267 (25.4)	24 (24.7)	30 (28.3)	63 (21.7)	—
	Level 3	415 (39.5)	38 (39.2)	42 (39.6)	89 (30.7)	—
Family history of AD	No	319 (30.4)	36 (37.1)	40 (37.7)	136 (46.9)	<0.0001
	Yes	731 (69.6)	61 (62.9)	66 (62.3)	154 (53.1)	—
APOE*ϵ*4 carrier	No	656 (62.5)	68 (70.1)	73 (68.9)	168 (57.9)	0.078
	Yes	394 (37.5)	29 (29.9)	33 (31.1)	122 (42.1)	—
pTau/A*β*	≤0.024	906 (87.5)	74 (77.9)	71 (71.0)	167 (59.4)	<0.0001
	>0.024	130 (12.5)	21 (22.1)	29 (29.0)	114 (40.6)	—
MTA average	<1	856 (82.1)	63 (67.0)	75 (71.4)	158 (54.7)	<0.0001
	≥1	186 (17.9)	31 (33.0)	30 (28.6)	131 (45.3)	—
Fazekas scale deep (FSD)	<2	893 (85.7)	83 (88.3)	76 (72.4)	236 (81.7)	0.002
	≥2	149 (14.3)	11 (11.7)	29 (27.6)	53 (18.3)	—
Fazekas scale periventricular (FSPV)	<1	660 (63.3)	61 (64.9)	53 (50.5)	155 (53.6)	0.002
	≥1	382 (36.7)	33 (35.1)	52 (49.5)	134 (46.4)	—
ARWMC basal ganglia	<1	929 (89.2)	83 (88.3)	90 (85.7)	246 (85.1)	0.248
	≥1	113 (10.8)	11 (11.7)	15 (14.3)	43 (14.9)	—
ARWMC frontal	<1	346 (33.2)	36 (38.3)	27 (25.7)	86 (29.8)	0.182
	≥1	696 (66.8)	58 (61.7)	78 (74.3)	203 (70.2)	—
ARWMC infratentorial	<1	988 (94.8)	87 (92.6)	96 (91.4)	266 (92.0)	0.195
	≥1	54 (5.2)	7 (7.4)	9 (8.6)	23 (8.0)	—
ARWMC parieto-occipital	<1	549 (52.7)	48 (51.1)	45 (42.9)	127 (43.9)	0.025
	≥1	493 (47.3)	46 (48.9)	60 (57.1)	162 (56.1)	—
ARWMC temporal	<1	863 (82.8)	77 (81.9)	78 (74.3)	223 (77.2)	0.043
	≥1	179 (17.2)	17 (18.1)	27 (25.7)	66 (22.8)	—
ARWMC combined	<3	815 (78.2)	71 (75.5)	72 (68.6)	211 (73.0)	0.061
	≥3	227 (21.8)	23 (24.5)	33 (31.4)	78 (27.0)	—

On examining possible associations of longitudinal changes in volumetric imaging measures and the latent phenotype classes, we observe an increasing annualised hippocampal shrinkage with increasing class, without a similar trend being seen between annualised ventricular enlargement and latent phenotype class. Notably, latent phenotype class 2 had the largest annualised increase in ventricles volume (Table 3).

### 3.3 Neuropathological Endotypes—Results From Profile Regression

For the profile regression analysis, which links CSF and neuroimaging biomarkers to the latent clinical trajectory phenotype, we ran six independent MCMC chains, and obtained a posterior similarity matrix and associated PAM “representative” clustering from the output of each chain (see Section 2.2.2 for details). Agreement between these six chains was high with mean pairwise Pearson’s correlation of 0.95 (standard deviation 0.03) between the dissimilarity matrices and mean pairwise adjusted Rand index of 0.90 (standard deviation 0.05) between the representative clusterings. This, together with inspection of posterior parameters for each chain, suggests that there is no strong evidence against convergence of the MCMC and there is a good level of robustness of the clustering structure. Three of the “representative” clusterings have six clusters, while the other three had seven.

We present below the results of applying consensus clustering to aggregate the output from the six MCMC chains. The mean Pearson’s correlation between the consensus dissimilarity matrix and the six independent chains’ dissimilarity matrices was 0.98 (standard deviation 0.01). Similarly, the mean adjusted Rand index between the consensus representative clustering and the six representative clusterings from each chain was 0.93 (standard deviation 0.03). The final consensus representative clustering has seven clusters.

**FIGURE 3:**
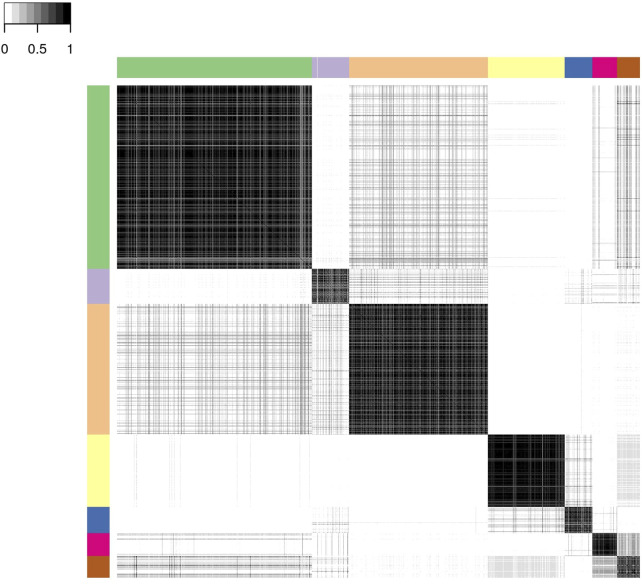
Posterior similarity matrix for the consensus across the six MCMC chains from the Bayesian profile regression analysis on the 1,543 EPAD participants. Each entry (*i*, *j*) of this 1,543 × 1,543-matrix represents the proportion of times participants *i* and *j* are assigned to the same cluster over the 250,000 × 6 MCMC iterations. Color bars indicate the seven final PAM consensus representative clusters of participants identified. See Figure 4 for more information regarding these clusters.

**FIGURE 4:**
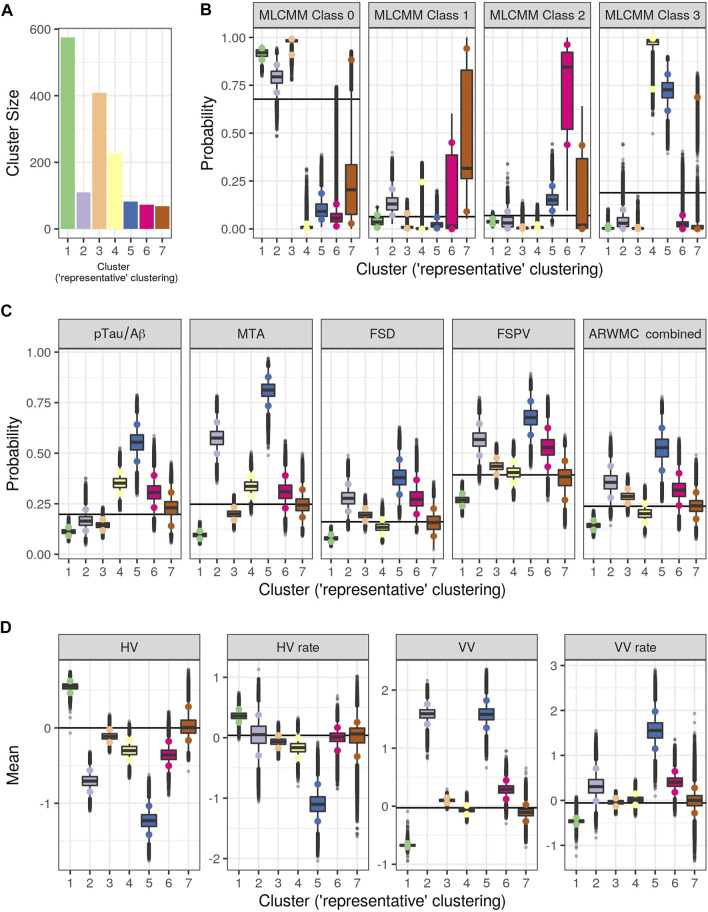
Results from Bayesian Profile Regression analysis. **(A)** Cluster sizes for the final PAM representative consensus clusters. **(B–D)** Posterior distributions for mean mixture component parameter values for each of the “representative” clusters (see Section 2.2.2). **(B)** Outcome variable (parameters are the probability of belonging to each MLCMM latent class). **(C)** Binary covariates (parameters are the probability of the covariate having value of one). **(D)** Continuous covariates (parameters are the mean covariate value). For **(A–D)**, colors indicate clusters (see also Figure 3). For **(B–D)**, black horizontal lines indicate the mean parameter values across all subjects and the coloured circles indicate the upper and lower limit of the 90% credible interval.

Figure 3 shows the consensus posterior similarity matrix that summarises the output from across the six MCMC chains and the seven representative clusters that were identified from this matrix. Figure 4 and Table 4 describe these seven clusters and their distinct biomarker profiles (i.e., neuropathological endotypes). Cluster 1, which is the largest cluster (comprising of 575 out of 1,543 participants), estimated the posterior mean probability of belonging to latent phenotype class 0 to be 92% (in agreement with the empirical estimate of 94%). It was characterised by participants with lower than expected/average probabilities of having abnormal pathology on the various biomarkers and above average healthy indicators of baseline and longitudinal volumetric measures for hippocampus and ventricles. We label this cluster as a “healthy brain” neuropathological endotype. It had on average the youngest participants, with a mean age (SD) of 61.4 (6.2) years.

**TABLE 4:** Results from the Bayesian profile regression analysis on the 1,543 participants.

		Posterior means												
		Probability of abnormal pathology					SD distance from overall mean				Class membership probability			
Clusters	N (%)	pTau/A*β*	MTA	FSD	FSPV	ARWMC combined	Mean HV	Mean HV rate	Mean VV	Mean VV rate	Class 0	Class 1	Class 2	Class 3
1	575 (37.3)	0.113	0.096	0.079	0.269	0.143	0.549	0.362	−0.674	−0.463	0.917	0.040	0.038	0.005
2	110 (7.1)	0.166	0.575	0.278	0.567	0.357	−0.706	0.047	1.590	0.321	0.791	0.133	0.038	0.039
3	409 (26.5)	0.145	0.200	0.195	0.436	0.287	−0.111	−0.066	0.101	−0.042	0.976	0.015	0.006	0.003
4	227 (14.7)	0.353	0.337	0.135	0.405	0.202	−0.300	−0.166	−0.063	0.020	0.010	0.040	0.009	0.941
5	82 (5.3)	0.553	0.810	0.380	0.675	0.524	−1.229	−1.093	1.583	1.558	0.101	0.024	0.154	0.721
6	72 (4.7)	0.308	0.309	0.276	0.529	0.319	−0.354	0.000	0.287	0.407	0.064	0.177	0.731	0.028
7	68 (4.4)	0.228	0.247	0.157	0.376	0.240	0.025	0.026	−0.109	−0.004	0.248	0.464	0.173	0.115
Overall empirical mean		0.194	0.247	0.158	0.393	0.236	5,793	-23.8	32,997	1,274	0.680	0.063	0.069	0.188
SD							705	88.7	17,687	1,264				

Cluster 2, which is a mixture of participants from both latent phenotype classes 0 and 1 (85 and 15% respectively), had somewhat lower than average AD positivity risk (but within the margin of uncertainty of the overall mean) and had stable hippocampal volume over time, but otherwise had higher than expected risk of abnormal pathology on the other biomarkers, including medial temporal lobe atrophy (MTA) indicating hippocampal involvement, and 1.59 standard deviations (SDs) higher baseline ventricles volume and 0.32 SD faster annual rate of increase in ventricles volume above their average, which is being tolerated so far. This cluster appears to be a non-AD driven cluster with “kindling” cerebrovascular disease. We label it as an “at-risk-of-vascular dementia” neuropathological endotype. The mean age (SD) of participants was 69.1 (7.0) years and percentage APOEe4 positive was 28%, the lowest amongst the clusters.

Cluster 3, which is the second largest in size (409 participants; all from latent phenotype class 0), is characterised by lower than expected risk of both AD positivity and MTA abnormal pathology and no clinically meaningful pathological indications on volumetric neuroimaging; but with evidence of white matter lesion pathology. We describe this cluster as a “healthy ageing” endotype especially as participants are on average older than those in cluster 1, with a mean age (SD) of 66.1 (6.5) years, and they appear to be able to compensate for some cerebrovascular disease. Moreover, none of the differences from overall average for any of the biomarkers were particularly large.

Cluster 4 has 15% of the participants—with average age (SD) of 68.1 (6.8) years–all of whom belong to latent phenotype class 3 (questionably cognitively impaired class). It is characterised by clinically meaningful increased risk of AD positivity and MTA abnormal pathology, early pathological indications on hippocampal volume markers and slightly increased proportion of APOE*ϵ*4 carriers relative to the overall average (0.4 versus 0.375) and may represent a subgroup of “AD high risk” participants.

Cluster 5 represents the 5% of the cohort who have the highest risk, worst baseline levels and fastest rate of worsening on markers. It comprises of a mixture of participants from latent phenotype classes 0 (6%), 2 (17%) and 3 (77%). We consider this to be an “AD-related cluster”. Moreover, it has the highest mean age of 74.2 years (SD 5.6 years) amongst the seven clusters and, notably, the highest proportion of APOE*ϵ*4 carriers (0.46) despite the EPAD selection mechanism.

Finally clusters 6 and 7, which are the most uncertain ones (i.e., empirical class membership proportions of 100% in class 2 for cluster 6 and class 1 for cluster 7 do not match with the corresponding mean posterior probabilities for these classes of 73 and 46% respectively), correspond to clusters where there are, respectively, evidence of increased abnormal pathology on all markers (except hippocampal atrophy and MTA) and no particular overall evidence of increased abnormal pathology beyond expected on any particular biomarker. Cluster 6 may be another AD-related cluster, but one, possibly, in an earlier stage of progression (cf cluster 5) as they are on average 5.6 years younger, with a mean age (SD) of 68.6 (6.3) years. Cluster 7 appears to have individuals with both unclear biomarker profiles and unclear cognitive trajectories, and therefore we describe it as an “ambiguous” cluster. The mean age (SD) here is 66.0 (6.5) years.

We assessed clustering validity through stability under repeated sub-setting (10 splits, totalling 20 subsets of the data). Out of the twenty consensus representative clustering structures obtained from applying Bayesian profile regression to the twenty subsets, 8 and 10 of these clustering structures consisted of four and five clusters respectively, while the other two comprised three and six clusters. Agreement between the consensus clusterings and the clusterings from the corresponding six independent MCMC chains across the twenty subsets were again high with mean adjusted Rand index of 0.93 (standard deviation of 0.10). The reduced number of clusters relative to the seven clusters found using the full data-set is likely due to the 50% reduction in sample size for the subsets. A comparison of the consensus representative clustering obtained using the subsets of data with the consensus representative clustering obtained using the full data-set (restricted to those individuals in each subset for the comparisons) resulted in a mean adjusted Rand index of 0.69 (standard deviation of 0.09). Furthermore, comparing the 20 consensus posterior dissimilarity matrices obtained from the subsets against those obtained using the corresponding submatrices of the full data-set resulted in a mean Pearson’s correlation of 0.860 (standard deviation of 0.049). These comparisons indicate good agreement between the results obtained on the subsets and those obtained using the full data-set, giving evidence for stability of our results.

Additionally, the held-out prediction analyses (training on one subset and predicting for the other in each split) resulted in a mean Pearson’s correlation of 0.674 (standard deviation of 0.045) between the predicted posterior dissimilarity matrices and the corresponding submatrices obtained using the full data-set. Comparing each of the estimated consensus representative clustering obtained from one subset of the split with the predicted clustering for this subset (predictions obtained using a model trained on the other subset of the data split only) resulted in a mean adjusted Rand index of 0.464 (standard deviation of 0.076) over the 20 comparisons. This performance on challenging held-out prediction tasks gives further support for the validity and stability of our clustering results.

The consensus representative clustering structure obtained using Bayesian profile regression without the MLCMM class outcome (i.e., only using biomarker covariates) had an adjusted Rand index of 0.48 with the clustering that did include the outcome, indicating that the outcome is playing an influential role in the clustering analysis and is facilitating interpretation of the clusters in terms of linking them to latent clinical phenotypes.

## 4 Discussion

In this paper, we demonstrate the usefulness of our two-stage approach in, firstly, characterising the evolution of correlated cognitive and clinical outcomes for LCS participants via an underlying latent process in which its trajectory depends on one of four latent clinical phenotypes, and then in providing biological insight through the identification of subgroups based on distinct biomarker profiles (i.e., neuropathological endotypes) linked to the latent phenotypes. Our approach recognises that the longitudinal cognitive and clinical outcomes are the downstream clinical manifestations/consequences of earlier endogenous biological changes occurring within the brain whether they be due to normal brain ageing or pathological due to a specific underlying disease process. It however does not attempt to assess the exact ordering of the pathological cascade of events.

Our intention here was not to provide a comprehensive clinical and biological investigation of the EPAD LCS data but to demonstrate the utility of our two-stage strategy in uncovering meaningful clinical and biological structure within this heterogeneous population. Therefore we chose to use a reduced set of coarser, but still relevant, ATN (amyloid-beta deposition (A), pathologic tau (T), and neurodegeneration (N)) and cerebrovascular biomarkers to demonstrate our two-stage approach. If interest lies in a more thorough investigation, then our approach can be extended to incorporate a larger set of biomarkers, providing more granular information (e.g., both left and right MTAs and hippocampal volumes and all five ARWMC regions could be considered instead of the average, total or majority as was done in this paper; with additional markers such as the Koedam score, which measures parietal atrophy, included), and additional correlated cognitive or clinical outcomes (e.g., specific cognitive domains). However, with more biomarkers being considered, this could result in increased uncertainty and instability in clustering structure obtained through use of Bayesian profile regression. Therefore we would recommend the incorporation of a variable selection component into the Bayesian profile regression analysis in order to identify the actual drivers of the clustering structure. Related issues may arise regarding both the number and relevance of latent classes arrived at when additional outcomes are added to the multivariate latent class mixed effects analysis, especially when weakly informative or conflicting outcomes are included.

The latent process arising from the multivariate latent class mixed modelling (MLCMM) approach appeared to be more highly correlated with the observed transformed CDR sum of boxes score than to the normalised Mini-Mental State Examination score, possibly reflecting the former being more sensitive to underlying changes than the latter early on. Nevertheless both CDRSB and MMSE produced concurring patterns with each other across the four latent phenotype classes (see Figure 2). These four trajectories correspond to a normal cognitive functioning class throughout, a reversion class, a declining class and a (questionable) cognitively impaired class. They are consistent with what has been reported previously in the literature, although the reversion class probably reflects measurement error. Interestingly, with our Bayesian profile regression analysis, we were able to find endotypes covering the full spectrum from “healthy brain” to “AD-related” within the EPAD cohort; reflecting one of the aims of the EPAD LCS to provide a well-phenotyped population covering the full continuum of risk of subsequent AD dementia development.

We note that the diminishing numbers at each visit reflect both the staggered opening of the 31 recruitment centres across Europe and the LCS concluding at the end of the IMI funding period. Attempts to further fund the cohort as a whole across Europe were not successful, in large part due to the COVID-19 pandemic. Attempts are ongoing to follow-up these participants in a series of studies across Europe to provide longer term clinical and biological outcomes.

The second objective of the EPAD LCS was to create a trial-ready cohort for potential recruitment into the EPAD PoC Trial. Unfortunately, this trial was not realised. However, our approach can still be used to demonstrate trial-readiness with respect to both minimising screen failures and identifying participants with particular biomarker profiles eligible for recruitment. For example, participants identified/pre-screened as belonging to the “healthy brain” or “healthy ageing” clusters would not be considered for inclusion into trials thereby reducing screen-failure rates currently seen in AD-related trials due to the low prevalence of AD pathology in individuals without dementia, especially among cognitively unimpaired. Whereas, for example, individuals in clusters 4, 5 or 6 may be specifically targeted for phase II trials in which volumetric neuroimaging biomarkers are used as “surrogate” endpoints. While secondary prevention trials in pre-clinical populations with no baseline cognitive impairment may be more inclined to focus recruitment on participants from cluster 6 (or class 2) when the primary endpoint is a cognitive one.

The novelty of our approach is not only in characterising the longitudinal cognitive and clinical outcomes into latent phenotype trajectories and in identifying neuropathological endotypes, but going beyond identifying substructures to also being able to do future longitudinal clinical prediction in individuals. Briefly, we would combine the posterior predictive probabilities of class membership obtained from both the Bayesian profile regression and the MLCMM, based on the observed relevant biomarker, cognitive and risk factor data, to update the individual’s mixture component probabilities in the MLCMM. We would then use these as weights to average over the linear mixed effects submodels corresponding to the four classes in order to predict future transformed CDRSB and normalised MMSE. Currently, the uncertainty attached to the latent trajectory classes is not taken account of in the Bayesian profile regression analysis in our two-stage approach, although this can be rectified by using Markov melding (Goudie et al., 2019). However, we expect this to have little impact on our findings.

In conclusion, we have introduced a two-stage approach for the modelling of longitudinal cognitive and clinical outcomes, biomarkers (baseline and longitudinal) and risk factors to analyse the data from the EPAD Longitudinal Cohort Study and shown its clinical and biological utility in the areas of trajectory stratification, subgroup identification and prediction. In the long term we envisage this approach to be applicable more widely to precision medicine and secondary prevention in Alzheimer’s dementia research and practice.

## Data Availability

A publicly available dataset was analyzed in this study. This dataset can be found at: http://ep-ad.org/erap/, doi: 10.34688/epadlcs_v.imi_20.10.30.
